# Supplementary material for: Keratin filament mechanics and energy dissipation are determined by metal-like plasticity
Source: Matter. 2023 Jun 7;6(6):2019–33. doi: 10.1016/j.matt.2023.04.014 (PMC10273143; doi:10.1016/j.matt.2023.04.014)
Supplement: Document S1. Figures S1–S3, Tables S1 and S2, and supplemental experimental procedures [file mmc1.pdf]

Matter, Volume *6*

Supplemental information

Keratin filament mechanics and energy dissipation are determined by metal-like plasticity

Charlotta Lorenz, Johanna Forsting, Robert W. Style, Stefan Klumpp, and Sarah Köster

Supplemental figures

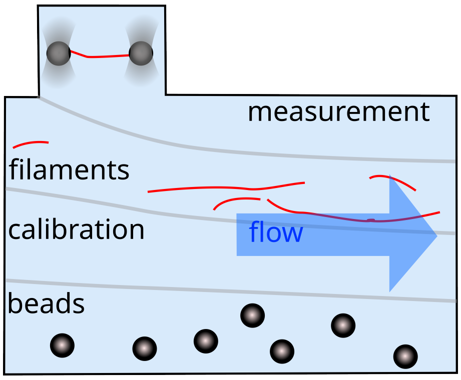

Figure S1: **Sketch of the microfluidic setup combined with optical tweezers to stretch single intermediate filaments.** The thin gray lines denote the borders of the different solutions flowing side-by-side in a laminar manner, without turbulent mixing.

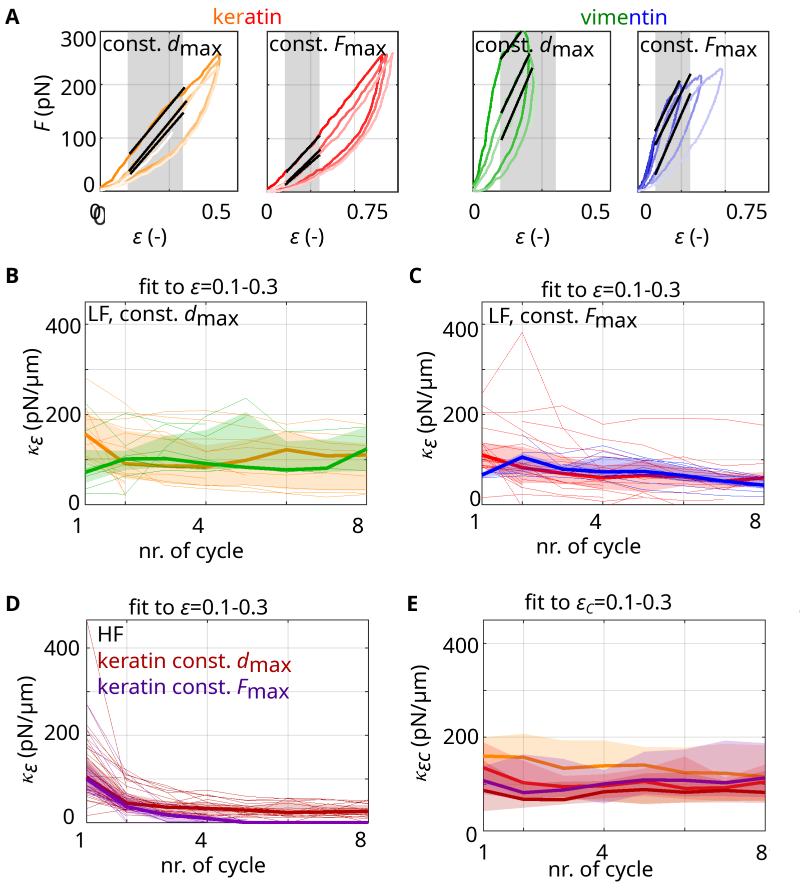

Figure S2: **Keratin filaments retain their stiffness upon repeated loading**, related to Fig. 3. (A) Typical experimental data sets of keratin (orange, red) and vimentin (green, blue) filaments stretched three times to a constant $d_{\max}$ (orange, green) and to a constant $F_{\max}$ (red, blue) including fits to $\varepsilon = 0.1 - 0.3$ (gray shaded area, solid black lines); same data as shown in Fig. 2A. (B,C) Keratin (orange, red) and vimentin (green, blue) filament stretching stiffness κ_ε resulting from the fits shown in a. (B) constant $d_{\max}$ with $F_{\max}$ in the LF range; (C) constant $F_{\max}$ in the LF range. (D) Keratin filament stiffness κ_ϵ derived from linear fits to $\varepsilon = 0.1 - 0.3$ for keratin filaments stretched to the HF range with a constant $d_{\max}$ (dark red) or to a constant $F_{\max}$ (purple). (E) Corrected $\kappa_{\epsilon c}$ for keratin filaments for all conditions studied: constant $d_{\max}$ with $F_{\max}$ in the LF range (orange), constant $F_{\max}$ in the LF range (red), constant $d_{\max}$ with a $F_{\max}$ in the HF range (dark red) and constant $F_{\max}$ in the HF range (purple). (B-E) Thick lines show the median and shading indicates the area between the 25th and 75th percentile of the distributions per cycle.

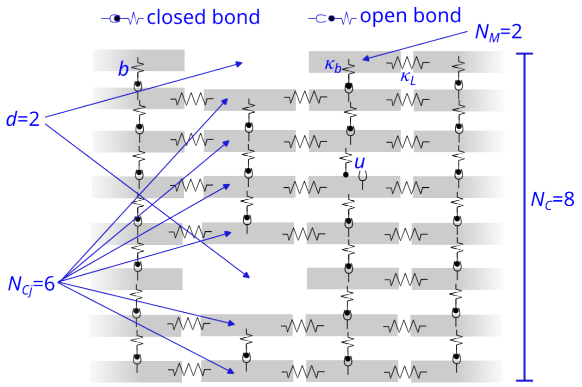

Figure S3: **Sketch of the model describing subunit sliding**, related to Fig. 4. The parameters are defined in the Supplemental experimental methods.

Supplemental tables

Variable	Meaning	Value
ΔG	Energy difference between a laterally bound and unbound subunit	6
κ_b	Stiffness of a lateral bond between two subunits	4.6
κ_L	Stiffness of all linkers between subunits	60
N_C	Number of subunits within a filament	8
N_F	Number of unit-length filaments within a filament	100
N_M	Number of monomers within a subunit	2
N_P	Number of parallel monomers in a unit-length filament	16
p_S	Probability that a slid dimer binds to the next dimer	10%
v	Loading speed	10000

Table S1: **Dimensionless parameters and values for simulation of single stretched keratin filaments.**

Variable	Meaning	Value
ΔG	Energy difference between a laterally bound and unbound subunit	2
κ_α	Stiffness of a lateral bond between two subunits	5.5
κ_β	Stiffness of all linkers between subunits	5.5
κ_L	Stiffness of all linkers between subunits	100
N_F	Number of unit-length filaments within a filament	100
N_P	Number of parallel monomers in a unit-length filament	32
v	Loading speed	2000

Table S2: **Dimensionless parameters and values for simulation of single stretched vimentin filaments.**

Supplemental experimental procedures

Materials and methods

Protein is expressed, labeled and dialyzed as described in Ref. 1. After assembly, keratin filaments are diluted 1:10 with keratin assembly buffer (10 mM TRIS, pH 7.5)[2–4] and stored at 37°C for up to two days. Vimentin filaments are stored in vimentin assembly buffer (100 mM KCl, 2 mM phosphate buffer, pH 7.5)[5,6] at 4°. For measurements with the optical tweezers setup (LUMICKS, Amsterdam, Netherlands), we load a four-channel microfluidic chip. Two maleimide-coated polystyrene beads (Kisker Biotech, Steinfurt, Germany) are captured with the optical tweezers from the beads channel and calibrated via the thermal noise spectrum. All filaments are stretched in vimentin assembly buffer, which corresponds to the high-ionic strength buffer condition used in Ref. 1.

All data analysis and image processing is carried out with self-written MatLab codes.

Simulations

Single keratin filaments under tension are modeled based on Refs. 1,7,8. One unit length filament (ULF) has N_P monomers, which are arranged in parallel and all have the same length before loading. The monomers can be divided into N_C N_M-mers, i.e., a keratin filament[9] has $N_C = 8$ dimers with $N_M = 2$. We refer to these N_M-mers as "subunits", which have the ability to slide with respect to each other. When subunits slide, the bonds between them open and re-form at the neighboring periodic position in the direction of the applied force as sketched in Fig. 4B. In accordance with previous findings[10,11] we assume that interactions within subunits are strong compared to interactions between these subunits so that the interactions within subunits, i.e. between monomers, do not break. Yet, the model is still valid if subunits are assumed to be

equivalent monomers. Once a dimer has slid, it either pulls all dimers in a protofilament with it or it leaves a defect, i.e., an empty dimer as sketched in Fig. 4A, B.

We model the interaction between subunits by a spring with a spring constant κ_b and by an element that can open into an unbound state u when force is applied and can close again into a bound state b, when the connection to a new dimer forms. A sketch of the model is shown in Fig. S3. B_j is the number of intact lateral bonds between dimers in the jth ULF. The longitudinal interaction between subunits forming a protofilament is described with a spring constant κ_L.

Thus, the spring constant of the jth ULF is:

$$\kappa_j = N_C \left(\frac{1}{\kappa_L} + \frac{1}{B_j \kappa_b} \right)^{-1} .$$

The spring constant of the entire filament is calculated by $\kappa_F = 1/\left(\sum_{j=1}^{N_F} 1/\kappa_j\right)$, where N_F is the number of ULFs in a filament. Similar to Ref. 7, we neglect viscous and entropic contributions for simplicity.

We assume that the sliding of the subunits is a close-to-equilibrium process under force. The equilibrium reaction constant K_{eq} for subunits to unbind is:

$$K_{eq} = \frac{r^{b \to u}}{r^{u \to b}} = \exp(-\Delta G/(k_B T)) = 1/\gamma \,, \tag{1}$$

where we define $\gamma = \exp(\Delta G/(k_B T))$.

The force is distributed among the number of independent subunits N_C. The effect of the force on the rates is distributed between the binding and unbinding rates with a load distribution factor[12,13] θ, which ensures detailed balance and thus that the model leads to a thermodynamic equilibrium state in the non-driven limit.

The force $\phi = F/F_b$ is dimensionless and scaled to the force F_b which is required to open a bond between two subunits. The time τ is dimensionless and related to the time t with the zero-force reaction rate from a subunit binding to another subunit to the unbound state $r_0^{b \to u}$ by $\tau = r_0^{b \to u} t$. We assume Bell-Evans kinetics[14], so that the bound to unbound transition rate is:

$$r_{B_j}^{b \to u} = B_j r_0^{b \to u} \exp\left(\frac{\theta \phi}{B_j} \right) . \tag{2}$$

In this study, we set $\theta = 1$, so only the unbinding rate is affected by the force. We assume that there is no restoring force acting on subunits which rebind to a neighboring subunit in the direction of loading as sketched in Fig. 4A, B. Yet, subunits can unbind without rebinding to the next subunit in the direction of loading because the disposition of the dimer is not sufficient to displace the subunit up to the periodic position. To describe the rebinding event to a new subunit or rebinding to the original subunit, we calculate the rebinding rate $r_{B_j}^{u \to b}$ depending on the force distributed on the N_{Cj} subunits of the jth ULF (if the ULF possesses d defects, the number of subunits $N_{Cj} = N_C - d$):

$$r_{B_j}^{u \to b} = r_0^{b \to u} \gamma \exp\left(\frac{-(1-\theta)\phi}{N_{Cj}} \right) \tag{3}$$

We assume that the filament possesses 50 defects before it is stretched to approximate the experimental data. From these rates, we calculate the probability P_{B_j} that a certain number of subunits is bound laterally to another subunit. The dynamics of this quantity are given by a master equation which we simulate using the Gillespie algorithm:

$$\begin{aligned} \frac{dP_{B_j}}{dt} &= r_{B_j+1}^{b \to u} P_{B_j+1} + r_{B_j-1}^{u \to b} P_{B_j-1} \\ &\quad -(r_{B_j}^{b \to u} + r_{B_j}^{u \to b}) P_{B_j} . \end{aligned} \tag{4}$$

When a subunit unbinds, the ULF extends by ΔL and with a probability of $p_S = 10\%$ the dimer slides and can bind to a new dimer to approximate the experimental data. If the filament is not stretched further than an effective elongation of 0.15, dimers do not slide and only unbind

and rebind at the same position. We calculate the effective elongation by analyzing the extension of the filament during a retraction cycle at a force of 0.5 (arbitrary units).

To make the simulation run dimensionless, we normalize $\lambda = \Delta L/L_1$, where L_1 is a characteristic length of a unit-length filament. Thus, in this case, the extension of the jth ULF λ_j is:

$$\lambda_j = \begin{cases} 0 & \text{if } B_j > 0 \\ 1 & \text{if } B_j = 0. \end{cases} \tag{5}$$

The total extension of the filament then is $\lambda_{tot} = \sum_{i=1}^{N_F} \lambda_j$. Since the optical traps pull on the filament with a constant velocity v, the end-to-end distance is $x(t) = vt$. The force on the filament becomes

$$\phi = \kappa_F(x - \lambda_{tot})\,. \tag{6}$$

To simulate the data shown in Fig. 5, we use the parameters provided in Table S1.

The simulation of single stretched vimentin filaments is taken from Ref. 7 with the parameters provided in Table S2.

To convert these parameters into real values with actual units, fitting of the resulting force-strain curves would be necessary as shown in the Supplemental Material of Ref. 1.

Supplemental references

[1] Lorenz, C., Forsting, J., Schepers, A. V., Kraxner, J., Bauch, S., Witt, H., Klumpp, S., and Köster, S. (2019). Lateral subunit coupling determines intermediate filament mechanics. Phys. Rev. Lett. 123, 188102. 10.1103/PhysRevLett.123.188102.

[2] Coulombe, P. A. and Fuchs, E. (1990). Elucidating the early stages of keratin filament assembly. J. Cell Biol. 111, 153–169. 10.1083/jcb.111.1.153.

[3] Herrmann, H., Häner, M., Brettel, M., O Ku, N., and Aebi, U. (1999). Characterization of distinct early assembly units of different intermediate filament proteins. J. Mol. Biol. 286, 1403–20. 10.1006/jmbi.1999.2528.

[4] Herrmann, H., Wedig, T., Porter, R. M., Lane, E. B., and Aebi, U. (2002). Characterization of early assembly intermediates of recombinant human keratins. J. Struct. Biol. 137, 82–96. 10.1006/jsbi.2002.4466.

[5] Mücke, N., Wedig, T., Bürer, A., Marekov, L. N., Steinert, P. M., Langowski, J., Aebi, U., and Herrmann, H. (2004). Molecular and biophysical characterization of assembly-starter units of human vimentin. J. Mol. Biol. 340, 97–114. 10.1016/j.jmb.2004.04.039.

[6] Kretsinger, R. H., Uversky, V., and Permyakov, E. (2013). Encyclopedia of Metalloproteins (Springer New York).

[7] Block, J., Witt, H., Candelli, A., Danes, J. C., Peterman, E. J., Wuite, G. J., Janshoff, A., and Köster, S. (2018). Viscoelastic properties of vimentin originate from nonequilibrium conformational changes. Sci. Adv. 4, eaat1161. 10.1126/sciadv.aat1161.

[8] Forsting, J., Kraxner, J., Witt, H., Janshoff, A., and Köster, S. (2019). Vimentin intermediate filaments undergo irreversible conformational changes during cyclic loading. Nano Lett. 19, 7349–7356. 10.1021/acs.nanolett.9b02972.

[9] Herrmann, H., Häner, M., Brettel, M., Müller, S. A., Goldie, K. N., Fedtke, B., Lustig, A., Franke, W. W., and Aebi, U. (1996). Structure and assembly properties of the intermediate filament protein vimentin: the role of its head, rod and tail domains. J. Mol. Biol. 264, 933–953. 10.1006/jmbi.1996.0688.

[10] Premchandar, A., Kupniewska, A., Tarnowski, K., Mücke, N., Mauermann, M., Kaus-Drobek, M., Edelman, A., Herrmann, H., and Dadlez, M. (2015). Analysis of distinct molecular assembly complexes of keratin k8 and k18 by hydrogen–deuterium exchange. J. Struct. Biol. 192, 426 – 440. 10.1016/j.jsb.2015.10.001.

[11] Premchandar, A., Mücke, N., Poznański, J., Wedig, T., Kaus-Drobek, M., Herrmann, H., and Dadlez, M. (2016). Structural dynamics of the vimentin coiled-coil contact regions involved in filament assembly as revealed by hydrogen-deuterium exchange. J. Biol. Chem. 291, 24931–24950. 10.1074/jbc.M116.748145.

[12] Kolomeisky, A. (2015). Motor Proteins and Molecular Motors (CRC Press).
[13] Fisher, M. E. and Kolomeisky, A. B. (1999). The force exerted by a molecular motor. Proc. Natl. Acad. Sci. USA 96, 6597–6602. 10.1073/pnas.96.12.6597.
[14] Bell, G. (1978). Models for the specific adhesion of cells to cells. Science 200, 618–62. 10.1126/science.347575.
