# Supplementary material for: Keratin filament mechanics and energy dissipation are determined by metal-like plasticity
Source: Matter. 2023 Jun 7;6(6):2019–33. doi: 10.1016/j.matt.2023.04.014 (PMC10273143; doi:10.1016/j.matt.2023.04.014)
Supplement: Document S2. Article plus supplemental information [file mmc3.pdf]

Matter

CellPress
OPEN ACCESS

Article

Keratin filament mechanics and energy dissipation are determined by metal-like plasticity

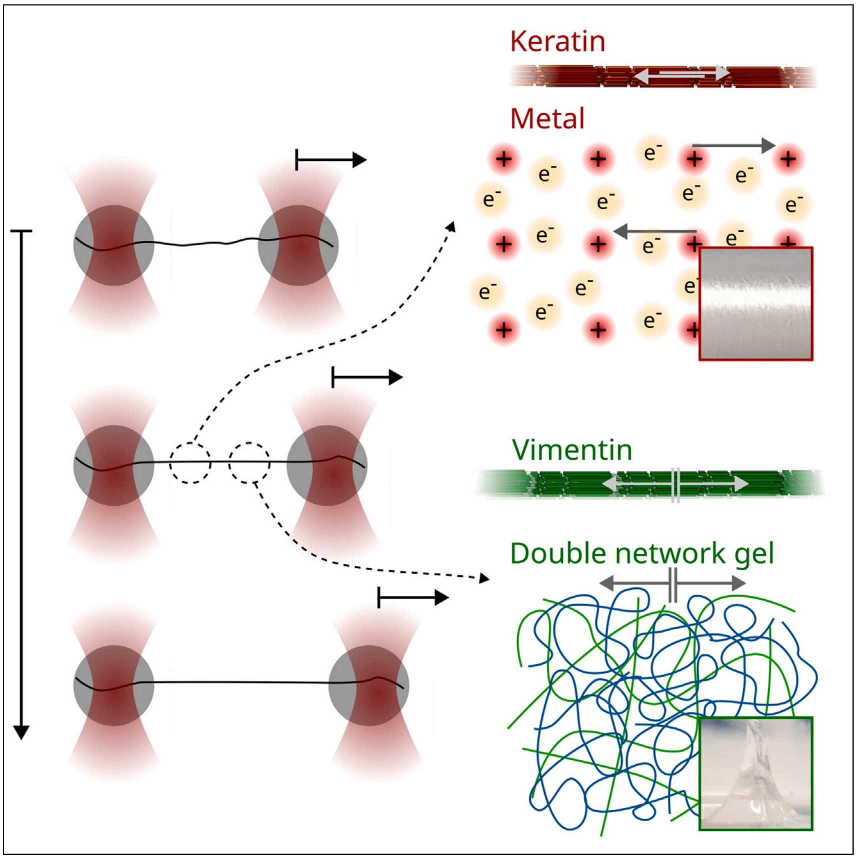

Charlotta Lorenz, Johanna Forsting, Robert W. Style, Stefan Klumpp, Sarah Köster

sarah.koester@uni-goettingen.de

Highlights

Keratin and vimentin filaments dissipate over 50% of supplied energy

Energy dissipation is based on very different physical mechanisms

Keratin filaments elongate but do not soften when repeatedly pulled

Vimentin filaments soften but do not elongate when repeatedly pulled

We mechanically characterize two different biological protein materials that exhibit softening and elongation properties reminiscent of solid metals and dual network gels, respectively. Both materials can also dissipate large amounts of energy. These very different material properties originate from minor structural differences between the proteins, therefore offering new strategies for the development of switchable materials with a unique portfolio of mechanical properties.

3 Understanding
Dependency and conditional studies on material behavior

Lorenz et al., Matter 6, 2019–2033
June 7, 2023 © 2023 The Authors. Published by Elsevier Inc.
https://doi.org/10.1016/j.matt.2023.04.014

Matter

CellPress
OPEN ACCESS

Article

Keratin filament mechanics and energy dissipation are determined by metal-like plasticity

Charlotta Lorenz,[1,2] Johanna Forsting,[1] Robert W. Style,[2] Stefan Klumpp,[3,4] and Sarah Köster[1,4,5,*]

SUMMARY

Cell mechanics are determined by an intracellular biopolymer network, including intermediate filaments that are expressed in a cell-type-specific manner. A prominent pair of intermediate filaments are keratin and vimentin, as they are expressed by non-motile and motile cells, respectively. Therefore, the differential expression of these proteins coincides with a change in cellular mechanics and dynamic properties of the cells. This observation raises the question of how the mechanical properties already differ on the single filament level. Here, we use optical tweezers and a computational model to compare the stretching and dissipation behavior of the two filament types. We find that keratin and vimentin filaments behave in opposite ways: keratin filaments elongate but retain their stiffness, whereas vimentin filaments soften but retain their length. This finding is explained by fundamentally different ways to dissipate energy: viscous sliding of subunits within keratin filaments and non-equilibrium α helix unfolding in vimentin filaments.

PROGRESS AND POTENTIAL

Nature offers a large portfolio of high-performance materials that can serve as a blueprint for novel sustainable materials and are used, for example, in biomedicine, industrial applications, and soft robotics. In particular, their distinct adaptability and non-linear material properties are of great interest for the design of biologically inspired materials.

We have identified two types of protein filaments—keratin and vimentin—that are exceptionally stretchable and can dissipate a large fraction of any supplied energy. These properties make them excellent candidates for shock-absorbing materials. Due to minor structural differences, they exhibit opposite behavior when stretched: keratin filaments stretch with each pull and maintain their stiffness (like solid metals), while vimentin filaments soften but maintain their length (like double-network gels).

INTRODUCTION

Biological cells possess an astounding composite materials system, the so-called cytoskeleton, which ensures mechanical integrity and stability and is responsible for active processes such as cell division and migration. Three families of biopolymers—actin filaments, microtubules, and intermediate filaments—together with passive cross-linkers and active molecular motors form interpenetrating networks within cells,[1,2] which adapt precisely to the mechanical needs and functions of each cell type. In contrast to actin and tubulin, intermediate filament proteins are expressed in a cell-type-specific manner,[3–5] making them ideal candidates for cells to adapt their mechanical properties.[6] A prominent example of differential expression of intermediate filament proteins is the epithelial-to-mesenchymal transition,[7–13] which occurs during cancer metastasis, embryogenesis,[14] and wound healing.[15] These processes have in common that stationary, strongly interconnected epithelial cells change their phenotype to highly motile mesenchymal cells. Interestingly, epithelial cells typically express the intermediate filament protein keratin, whereas mesenchymal cells express vimentin.

We have recently shown that already on the single filament level keratin 8/18 filaments are softer and exhibit a very different force-strain behavior than vimentin filaments[16]: keratin filaments exhibit a nearly linear increase in force up to a strain of 0.7, after which filaments stiffen. Vimentin filaments are stiffer for strains for up to 0.15 and exhibit a plateau-like regime for strains between 0.15 and 0.8 in which the force barely increases. For strains larger than 0.8, vimentin filaments stiffen as well. These mechanical properties of intermediate filament proteins are closely related to their molecular architecture[17–19]: the monomers consist of three α-helical

This is an open access article under the CC BY license (http://creativecommons.org/licenses/by/4.0/).

domains flanked by intrinsically disordered head and tail domains, as sketched in Figures 1A and 1B.[20] In the case of keratin and vimentin filaments, 16 or 32 monomers, respectively, associate laterally in a stepwise manner to form unit-length filaments (ULFs).[3,20,21] ULFs associate longitudinally to form filaments, thus resulting in an array of monomers arranged laterally and longitudinally and connected by electrostatic and hydrophobic interactions.

It remains an open question whether the different force-strain behaviors of vimentin versus keratin filaments also lead to differing abilities to dissipate energy and absorb mechanical stress. In the case of vimentin filaments, the dissipative properties have already been well investigated: repeated stretching and filament relaxation experiment have shown that the α helices unfold during the first stretching and form random coils afterward, which are then cycled in length.[19] This non-equilibrium unfolding of α helices allows for energy dissipation of about 80% of the input energy.

Here, we compare stretch-relaxation cycles for vimentin and keratin filaments. We find that the filament types behave fundamentally differently: vimentin filaments soften but maintain their original length, whereas keratin filaments elongate but keep their original stiffness. Interestingly, both filament types are able to dissipate a large portion of the input energy but via completely different physical mechanisms. We model the mechanical properties of keratin filaments numerically and draw an analogy to the mechanical properties of metals, which, by means of delocalized electrons, are highly deformable at constant stiffness, although the material elongates when stretched.[22]

RESULTS

Keratin filaments elongate upon repeated loading

We previously showed that vimentin filaments, when repeatedly stretched and relaxed, do not plastically elongate but keep their original length.[18] Here, to compare this property in detail for keratin (K8/K18) and vimentin filaments, we employ an optical tweezers setup combined with a four-channel microfluidic chip and a confocal microscope as described in Lorenz et al.[16] The microfluidic chip is shown schematically in Figure S1. The chip contains four inlets leading to laminar side-by-side flow of (1) the bead solution, (2) the buffer solution for calibration of the optical traps, (3) the filaments in buffer, and (4) the buffer for performing the stretching experiments. First, two beads are captured with optical traps and moved to the calibration subchannel for determination of the trap stiffness. Next, the beads are moved to the filament subchannel and remain there until a single filament attaches to both beads with its two ends. The filament attached to the beads is then moved to the filament-free buffer subchannel and stretched by moving one of the beads using the respective optical trap. The elongation of the filaments upon stretching is determined via the displacement of this bead, and the applied force is calculated from the displacement of the second bead with respect to the trap center and the trap stiffness. We include measurements in our datasets that are associated with single filaments and exclude bundles. We repeatedly stretch single filaments assembled from purified protein to a constant maximum distance (d_{max}), as sketched in Figure 1C. As a consequence, the maximum force (F_{max}) decreases with each stretching cycle, i.e., over time (t; Figure 1D, left panels). In the plots, progressing t is indicated by lighter color. To be able to compare different filaments, independent of their individual length, we calculate the strain $\varepsilon = \Delta L / L_0$, i.e., we normalize the filament length gained by stretching $\Delta L = (L - L_0)$ by the original filament length L_0 at 5 pN.[16,17] Vimentin filaments are stretched to a d_{max} that

[1]Institute for X-Ray Physics, University of Göttingen, Friedrich-Hund-Platz 1, 37077 Göttingen, Germany

[2]Department of Materials, ETH Zürich, Vladimir-Prelog-Weg 1-5/10, 8093 Zürich, Switzerland

[3]Institute for the Dynamics of Complex Systems, University of Göttingen, Friedrich-Hund-Platz 1, 37077 Göttingen, Germany

[4]Max Planck School "Matter to Life", Friedrich-Hund-Platz 1, 37077 Göttingen, Germany

[5]Lead contact

*Correspondence: sarah.koester@uni-goettingen.de

https://doi.org/10.1016/j.matt.2023.04.014

CellPress
OPEN ACCESS

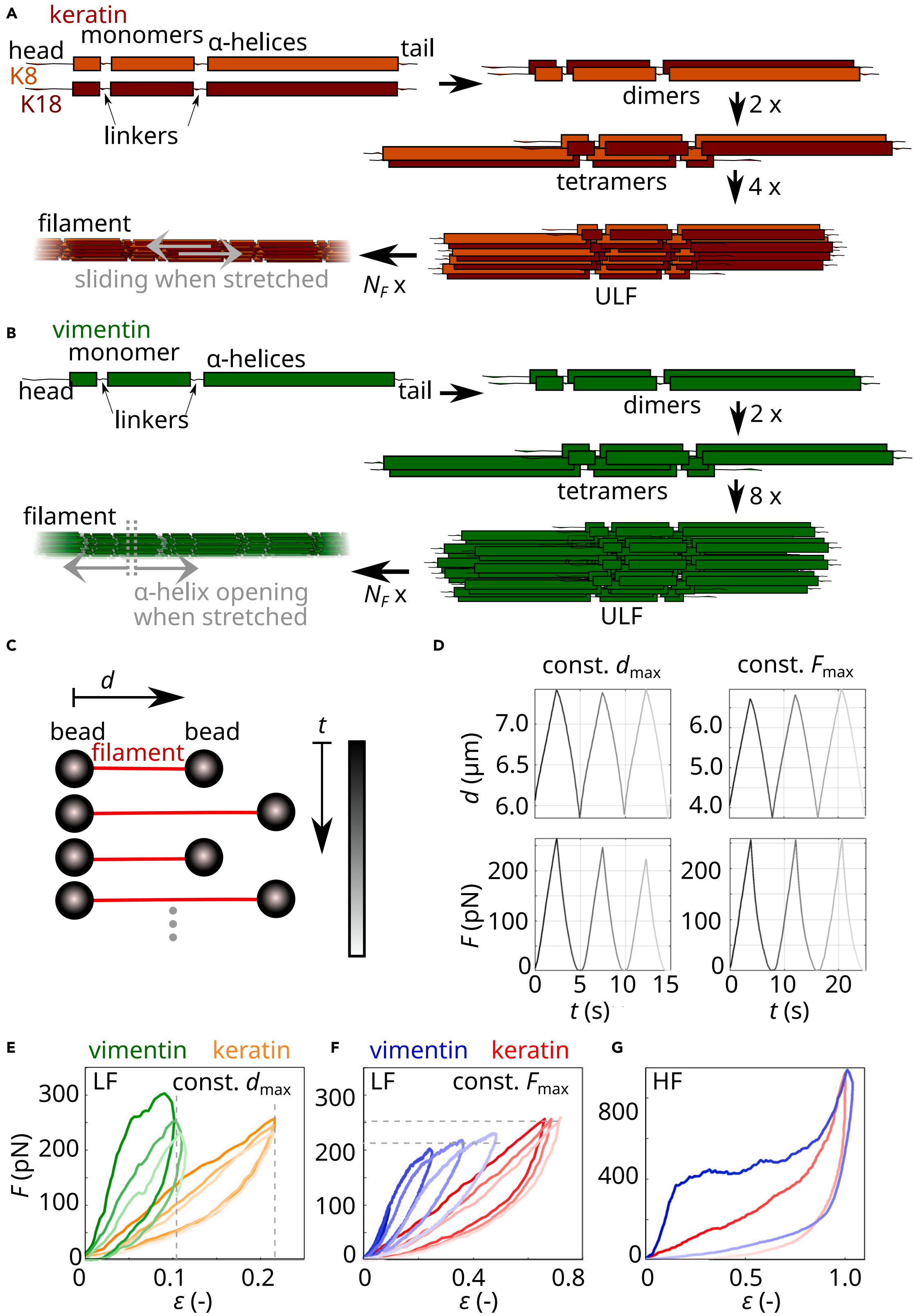

Figure 1. Filament assembly and experimental procedures

(A and B) Sketch of the assembly pathway of keratin intermediate filaments from keratin 8 (K8) and K18 (A) and of vimentin intermediate filaments (B).

(C) Sketch of the measurement procedure.

(D) Typical distance-time and force-time curves for constant d_{max} and decreasing F_{max} (left panels) and constant F_{max} measurements with an increasing d_{max} (right panels). Increasing time is indicated with lighter color.

(E–G) Typical force-strain curves of keratin (orange and red) and vimentin (green and blue) filaments when (E) repeatedly stretched to a constant d_{max} (dashed gray lines) in the low-force (LF) range, (F) repeatedly stretched to a constant F_{max} (dashed gray lines) in the LF range, and (G) stretched once to the high-force (HF) range and relaxed.

corresponds to the beginning of the plateau-like regime of the measured filament (green in Figure 1E). Thus, with this measurement protocol, we probe filament mechanics just before a majority of the α helices within vimentin filaments start to open.[17,18] Keratin filaments do not exhibit a plateau, so we fix d_{max} to the distance at which a force of 250 pN is reached during the initial cycle. A force of 250 pN is a typical force for the onset of the plateau when vimentin filaments are stretched.

To analyze whether the filaments are plastically deformed, i.e., elongated, during repeated stretching, we calculate the filament elongation by extrapolating linear fits to the elastic stretching regime of force-strain curves between $F = 100$ and 150 pN (gray shaded area), indicated by solid black lines in Figure 2A. The extrapolation of the linear fits to the x axis (dashed lines in Figure 2A) provides the effective length (ε_e) of the filament. This ε_e is calculated in units of strain and indicated by the solid circles. We observe that keratin filaments elongate by $\varepsilon_e = 0.1 - 0.2$ after eight stretching cycles, as shown in Figure 2B (orange), whereas vimentin filaments barely elongate (green).[19] The elongation of the keratin filaments is in line with our previous hypothesis of sliding subunits within these filaments.[16] Such sliding subunits do not experience a restoring force. On the contrary, vimentin filaments elongate only a little, as their subunits barely slide.

Our first measurement protocol, where we stretch the filaments to a fixed d_{max} for each cycle, corresponds to biological situations where cells are elongated to a constant distance. However, there are also physiological settings, such as during muscle contraction, during which cells are repeatedly stretched to a constant force, and we mimic this situation in our *in vitro* setting. Since keratin filaments do not exhibit a plateau-like regime, we chose a constant $F_{max} = 250$ pN that corresponds to the onset of the plateau-like regime in vimentin filaments, thus the onset of α helix unfolding. From a molecular point of view, this experimental protocol sheds light on the question of which mechanism within keratin filaments is instead responsible for elongation. We repeatedly stretch keratin and vimentin filaments to a constant F_{max} as shown in Figure 1D (right panel). Consequently, the d_{max} increases from one cycle to the next. Typical force-strain curves are shown in Figure 1F. We analyze the data in the same way as the data discussed so far, shown for keratin (red) and for vimentin (blue) in Figure 2A. Compared with measurements where filaments are stretched to a constant d_{max}, we find that keratin filaments elongate further (compare red data in Figure 2C with orange data in Figure 2B). The increased elongation is likely caused by a further d_{max} during the stretching cycles due to a constant F_{max}.

In specific biological situations, such as embryogenesis, cells may experience high forces and, consequently, high deformations. To include this high-force (HF) regime in our study, and to test whether keratin filaments elongate further with higher loading forces, we stretch keratin and vimentin filaments to up to 900 pN and beyond the plateau-like regime of vimentin filaments as shown in Figure 1G. Indeed, when stretched repeatedly to the HF range, keratin filaments elongate by

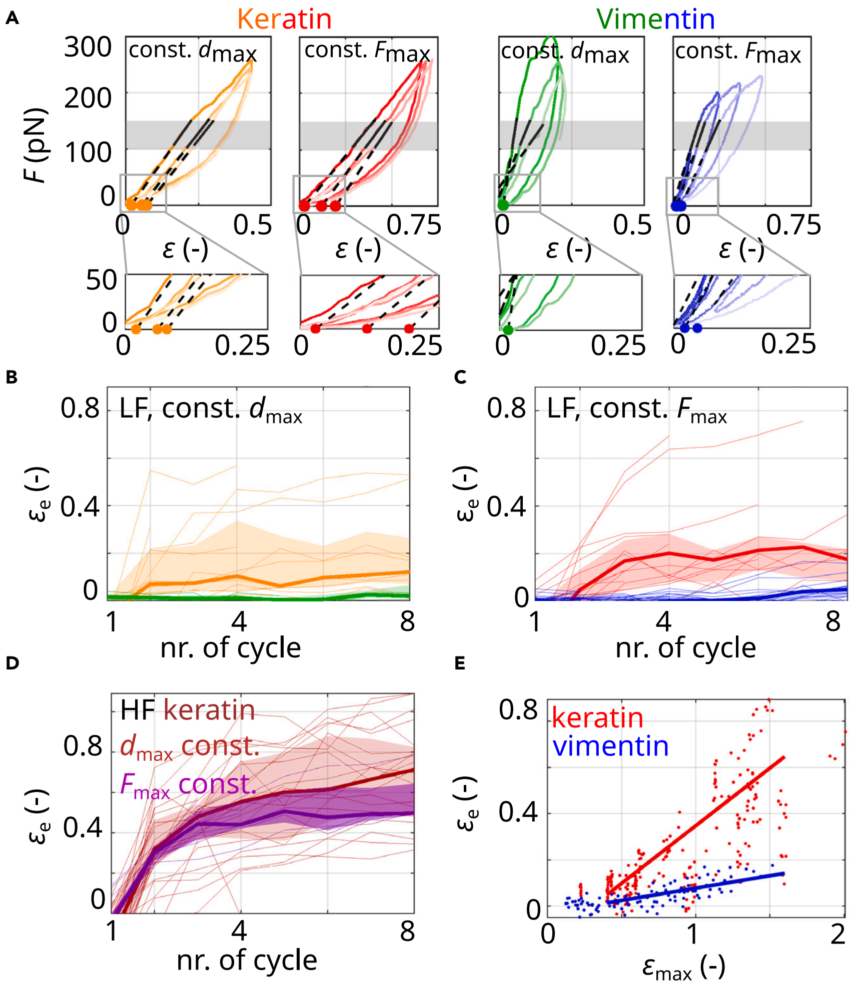

Figure 2. Keratin filaments elongate upon repeated loading

(A) Typical experimental datasets of keratin (orange and red) and vimentin (green and blue) filaments stretched three times to a constant d_{max} (orange and green) or to a constant F_{max} (red and blue) including fit curves to the force range of $F = (100 - 150)$ pN (solid black lines). Fit ranges are indicated by gray shaded areas. Filament elongations (solid circles on the x axis) are determined by extrapolation of the linear fits to the x axis (dashed lines).

(B and C) The effective length (ε_e) of filaments repeatedly stretched (B) to a constant d_{max} with F_{max} in the LF range or (C) to a constant F_{max} in the LF range.

(D) ε_e of keratin filaments stretched to a constant d_{max} with F_{max} in the HF range (dark red) or to a constant F_{max} in the HF range (purple).

(B–D) Thick lines show the median and shading indicates the area between the 25th and 75th percentiles of the distributions per cycle.

(E) ε_e of keratin (red) and vimentin filaments (blue) plotted against ε_{max} during the 4th to 15th cycles. The data are linearly fitted starting at a strain of 0.4 (solid lines).

$\varepsilon_e = 0.6 - 0.8$ after eight cycles, as shown in Figure 2D for a constant d_{max} (dark red) and a constant F_{max} (purple). To compare the different measurement protocols, we plot the ε_e of the cycles 4 to 15 against the maximum applied strain (ε_{max}) (see Figure 2E). Under the HF regime, both types of filaments elongate; however, keratin filaments (red) elongate further than vimentin filaments (blue). By fitting a linear relationship to the data above a strain of 0.4 (see red and blue solid lines in Figure 2E), we estimate that keratin filaments elongate around five times more at a given ε_{max} than vimentin filaments. In contrast to keratin filaments, vimentin filaments cannot

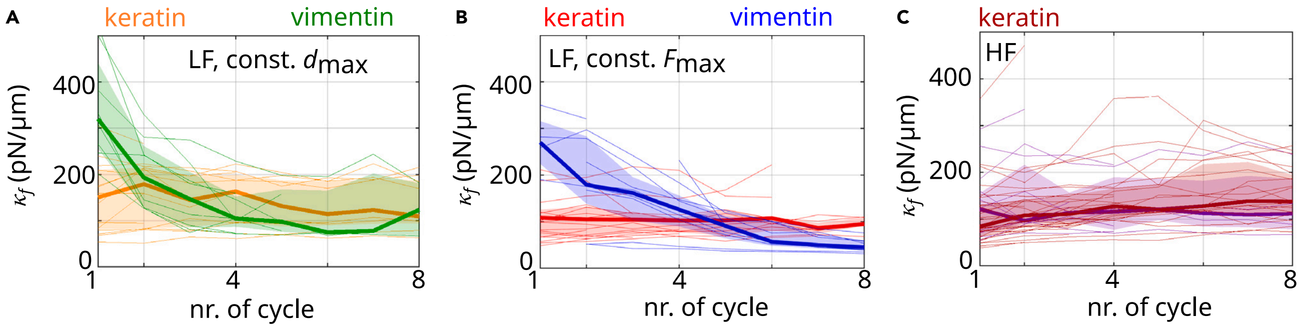

Figure 3. Keratin filaments retain their stiffness upon repeated loading

(A and B) Filament stiffness (κ_f) resulting from a fit to a force range of $F = (100 - 150)$ pN (gray shaded area in Figure 2A): (A) filaments stretched to a constant d_{max} (orange: keratin, green: vimentin), and (B) filaments stretched to a constant F_{max} (red: keratin, blue: vimentin).

(C) κ_f for keratin filaments stretched to a constant d_{max} in the HF range (dark red) and constant F_{max} in the HF range (purple).

(A–C) Thick lines show the median and shading indicates the area between the 25th and 75th percentiles of the distributions per cycle.

See also Figure S2.

be repeatedly stretched to similarly high forces, as they always rupture already during the second cycle. We attribute this behavior to structural changes during the first cycle that destabilize the filaments.

Keratin filaments maintain their stiffness upon repeated loading

The pronounced elongation of keratin filaments during repeated loading raises the question of how this elongation impacts filament mechanics. For vimentin filaments, it is known that structural changes occur during repeated stretching and that the filaments soften.[18,19] We characterize the filament mechanics by determining their stretching stiffness (κ_f) from the linear fits shown in Figure 2A. Remarkably, and in stark contrast to vimentin filaments,[18,19] keratin filaments retain their stiffness, no matter whether they are pulled to a constant d_{max} or constant F_{max} (orange and red in Figures 3A and 3B, respectively). The filament stiffness (κ_ε) can also be obtained from a linear fit to a defined strain range, e.g., 0.1–0.3, as shown in Figure S2A. This fit reveals a slight decrease of κ_ε for both keratin and vimentin filaments (see Figures S2B–S2D). However, since keratin filaments elongate, the fit range of the strain needs to be adjusted to the ε_e of the filament. We therefore calculate a corrected strain, $\varepsilon_c = \varepsilon - \varepsilon_e$, for each cycle. With this corrected strain, we confirm the result above that the stiffness of keratin filaments is constant (see Figure S2E). Thus, we can conclude that while keratin filaments are plastically deformed and that their length "remembers" the loading history, their stiffness "forgets" it. Vimentin filaments behave in exactly the opposite way: they have a tensile memory concerning stiffness and "forget" the loading history with respect to their lengths.[18,19] Our results are confirmed when keratin filaments are stretched to the HF regime. Here as well, keratin filaments retain their stiffness while elongating during repeated pulling. Figure 3C shows these data for a large d_{max} (dark red) or high F_{max} (purple).

An internal sliding mechanism explains the behavior of keratin filaments

The observation that the stiffness of keratin filaments is constant independent of the loading history and that the filament elongation depends on the loading history raises the question of which molecular mechanisms within keratin filaments cause this behavior that is so different from vimentin filaments. From repeated loading of vimentin filaments, we know that unfolded α helices do not directly transition to β sheets but turn into a third state, likely a random coil, which is softer than the α

CellPress
OPEN ACCESS

helices.[19] Thus, if a significant portion of the α helices within keratin filaments was unfolded, we would expect a softening of repeatedly stretched filaments since the softer subunits within the filament would be stretched first. However, we observe a constant stiffness, so we conclude that most α helices within the keratin filament remain intact. We do not exclude the possibility that a small portion of α helices unfold, but we hypothesize that these unfolded structures are not loaded during the next stretching cycle.

Thus, we suggest that the following molecular mechanism accounts for our findings: the subunits within keratin filaments slide and form new bonds at a different location with the same properties as the original location as a consequence of the periodicity of the structure. Thus, the stiffness during the next stretching cycle remains the same, and bonds with the same properties as the previously existing bonds are stretched. The proposed sliding mechanism is sketched in Figure 4A: filament subunits are represented by gray rectangles. We assume that these subunits are dimers.[23,24] These dimers are connected and interact as in a keratin filament *in vitro* (see Figure 1A). In the actual protein structure, these interactions could, for example, correspond to the knob-pocket mechanism suggested in Eldirany et al.[25] This mechanism also relies on a periodic structure, which would agree with our proposed periodic binding sites. Once a force is applied at $t_2 > t_1$, the dimers can slide, and new interactions can form (green arrows). The sliding of a longitudinally connected chain of dimers, i.e., a "protofilament," results in an elongation of the entire filament.[16] As this mechanism relies on the slippage of layers of periodically spaced units past each other, it is likely that it is similar to what is found in crystalline materials like metals or semi-crystalline polymers,[26] i.e., there are edge or screw dislocations in the keratin bundle that allow individual subunits to slide past each other—instead of requiring entire filaments to slip past each other in one step.

To show that this mechanism results in a constant stiffness, but further elongation of a filament upon repeated loading, we translate the suggested sliding mechanism into a computational model sketched in Figure 4B and described in detail in the supplemental information (supplemental experimental procedures; Figure S3; Tables S1 and S2). Our coarse-grained model takes into account fully assembled filaments, whereas existing atomistic simulations can capture stretched dimers or tetramers.[27,28] Interactions between dimers are represented by springs and elements, which can unbind under force. A breaking interaction between two dimers is represented by an unbinding of these elements. Upon application of force, the dimers can slide and rebind (cyan and magenta elements in Figure 4B). In our model, we assume that around 10% of dimers rebind to a neighboring dimer to approximate the experimental data. Sliding dimers, which do not bind to a neighboring dimer, can rebind to the dimer that they were originally bound to. This sliding and rebinding mechanism is highly similar to the sliding of metallic atoms in a metal upon force application as sketched in Figure 4C[29]: atoms are shown as black dots, and interactions due to delocalized electrons are represented by arrows between the atoms. When a force is applied, the atoms slide, and the electrons associate with a new atomic core (Figure 4C). Just as our suggested model for keratin filaments, this sliding mechanism also results in an elongation of metals and a constant stiffness under repeated loading.[29]

For vimentin filaments, the analogy to metals does not hold. Instead, they show a response similar to a stretched double-network hydrogel.[30] Strong bonds, corresponding to, e.g., covalent interactions (green squares in Figure 4F), connect the

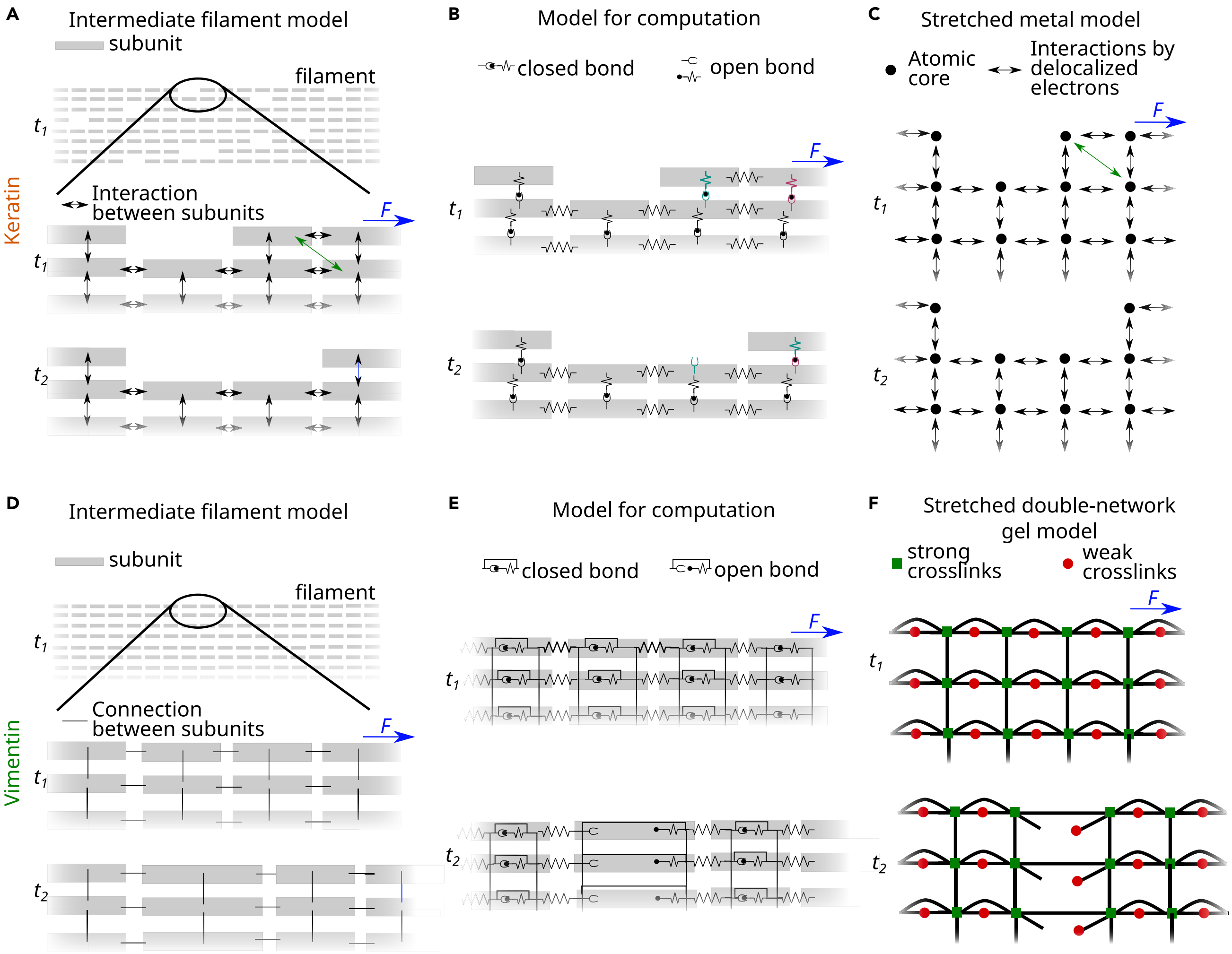

Figure 4. Stretched keratin filaments behave analogously to stretched metals, while stretched vimentin filaments behave analogously to a stretched double-network gel

(A) Sketch of the proposed sliding mechanism in keratin filaments. The gray rectangles represent subunits, i.e., dimers. The arrows between the rectangles indicate interactions. The green arrows indicate the formation of new interactions. When protofilaments slide, they cause an empty spot in the filament lattice, represented by an empty position without a gray rectangle.

(B) Sketch of the computational model to simulate force-extension curves of keratin filaments with the proposed sliding mechanism. The lateral interactions are modeled as elements that can either be in a closed or an open configuration. The longitudinal interactions are represented by springs. Under force at time $t_2 > t_1$, the lateral interactions (cyan and magenta at time t_1) break, and subunits slide to the periodic position, where they form new lateral bonds (cyan to magenta connection at t_2).

(C) Sketch of the sliding mechanism in bulk metals. The black circles represent atoms, and the arrows show delocalized electrons, which mediate the interactions between atoms. The shifted interactions are shown as green arrows.

(D) Sketch of the proposed sliding mechanism for vimentin filaments. Gray rectangles represent dimers. Black lines represent strong interactions between dimers. When stretched, the dimers are stretched and extend.

(E) Sketch of the computational model to simulate force-extension curves of vimentin filaments with the proposed opening of α helices. The α helices are modeled as elements that can either be in a closed or an open configuration. Lateral connections are represented by black lines. The longitudinal interactions are represented by springs. Under force at time $t_2 > t_1$, the α helices open, and the corresponding dimers elongate.

(F) Sketch of the mechanism in stretched double-network gels. Green squares represent strong cross-links and red circles weak cross-links, which are weaker than the strong cross-links so that they break first (compare t_2 and t_1).

See also Figure S3.

subunits laterally. Weaker bonds inside the dimers, i.e., opening α helices, corresponding to, e.g., ionic interactions (red circles in Figure 4F), lead to filament extension when stretched (see Figures 4D and 4E).[18] Indeed, double-network gels

CellPress
OPEN ACCESS

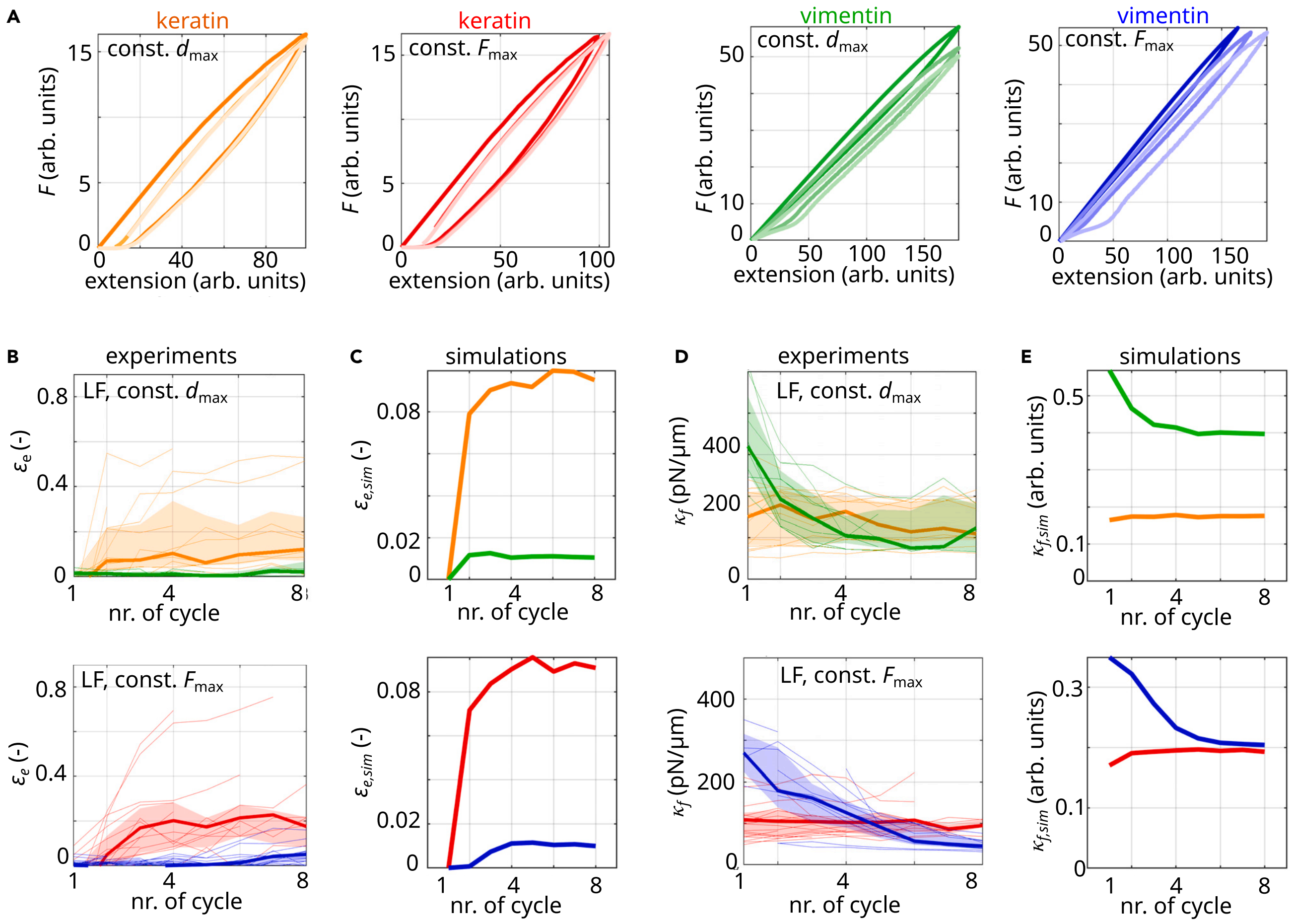

Figure 5. Internal sliding or unfolding mechanisms explain mechanical behavior of stretched keratin or vimentin filaments

(A) Simulated force-strain curves of repeatedly stretched keratin filaments (left panels, orange/red) and vimentin filaments (right panels, green/blue); lighter colors represent progressing time.

(B) Experimentally measured elongation (ε_e) of repeatedly stretched keratin and vimentin filaments (same data as in Figures 2B and 2C).

(C) Strain $\varepsilon_{e,sim}$ normalized by the maximum extension of stretched, simulated keratin filaments (orange and red) and stretched, simulated vimentin filaments (green and blue).

(D) Experimentally measured filament stiffness (κ_f) of repeatedly stretched keratin and vimentin filaments (same data as in Figures 3A and 3B).

(E) Stiffness $\kappa_{f,sim}$ of stretched, simulated keratin filaments (orange and red) and stretched, simulated vimentin filaments (green and blue).

show tough behavior upon single stretching but are weak when repeatedly stretched,[31] and this is exactly what we see in vimentin filaments. The two different elongation mechanisms for keratin and vimentin filaments are also indicated schematically in Figures 1A and 1B (light gray).

Running the computational model for keratin filaments shown in Figure 4B as a Monte Carlo simulation results in the force-extension curves shown in Figure 5A (left panels). For comparison, a vimentin filament under repeated extension is modeled with the simulation presented in Block et al.[18] (Figure 5A, right panels). We analyze the stiffness of the filaments and the elongation in the same way we analyze the experimental data. In excellent agreement with the experiments (Figures 5B and 5D), we find that keratin filaments extend (orange and red in Figure 5C), while vimentin filaments return to their original length (green and blue). Keratin filaments retain their stiffness (orange and red in Figure 5E) and vimentin filaments soften during repeated loading (green and blue). Thus, instead of α helix

unfolding as in vimentin filaments, subunits slide within keratin filaments and thereby avoid major α helix unfolding. Hence, next to cross-linkers,[19] subunit sliding can protect α helices from unfolding.

Keratin and vimentin filaments dissipate energy by different mechanisms

Vimentin filaments dissipate more than 80% of their input energy, and there are strong indications that this occurs by non-equilibrium α helix unfolding.[18] As keratin filaments do not possess this ability to unfold the α helices, the question remains if they dissipate a part of the input energy nevertheless, and, if so, by which mechanism. To investigate this phenomenon in detail, we analyze the dissipated energy during their first stretching and relaxation cycle as shown in Figures 6A and 6B as relative dissipated energy and absolute dissipated energy per filament length, respectively. We find that keratin filaments dissipate more than 50% of the input energy (red in Figure 6A), which is lower than for vimentin filaments (blue) but still a considerable amount. Figure 6B shows that in absolute units, both filament types dissipate energies on the order of 10^4 k_BT/μm. The high amount of dissipated energy supports the notion that keratin filaments may also act as cellular shock absorbers.[18,32,33]

Loading to higher forces (HF regime) also leads to high levels of relative energy dissipation (Figure 6C) with around 60%–70% of dissipated energy for keratin filaments (red) and 70%–80% of dissipated energy of vimentin filaments (blue). The absolute dissipated energy increases when stretched to higher forces, as shown in Figure 6D. These results apply to the first stretching cycle. Considering further stretching of the filaments, we find that vimentin filaments dissipate about twice as much energy as keratin filaments when repeatedly stretched (see Figure 6E).

Nevertheless, the question remains how energy is dissipated in keratin filaments on the molecular scale if it is not via the unfolding α helices. To dissipate energy, bonds need to be broken and must not rebind at the same position immediately. In the case of keratin filaments, our experiments indicate that most bonds within the α helices remain intact but that the bonds between subunits are broken. This can be seen from the data shown in Figure 2A, where the shape of the hysteresis loops does not change from cycle to cycle, suggesting that the molecular structure likely stays intact. Thus, we analyze the additional elongation ($\Delta\varepsilon_e$) from one cycle to the next compared with the dissipated energy during that cycle (see Figure 6F). $\Delta\varepsilon_e$ is a measure for the number of sliding dimers within the filament since dimer sliding causes elongation for keratin filaments. For keratin filaments (red in Figure 6F), higher relative dissipated energies are correlated with a more pronounced increase of $\Delta\varepsilon_e$. Since vimentin filaments barely elongate, we do not observe such a correlation between the dissipated energy and $\Delta\varepsilon_e$ (blue in Figure 6F). We therefore conclude that keratin filaments dissipate their energy by breaking bonds between subunits, which results in subunit sliding and filament elongation, whereas vimentin filaments dissipate their energy by α helix unfolding.[18]

DISCUSSION

Vimentin and keratin are an interesting pair of intermediate filaments, as the "switch" between them plays a major role in the epithelial-to-mesenchymal transition and, therefore, in cancer mestastasis, wound healing, and embryogenesis. Here, by repeated loading of single keratin filaments and comparing the results with vimentin cycling, we find surprisingly different behaviors: within keratin filaments, subunit

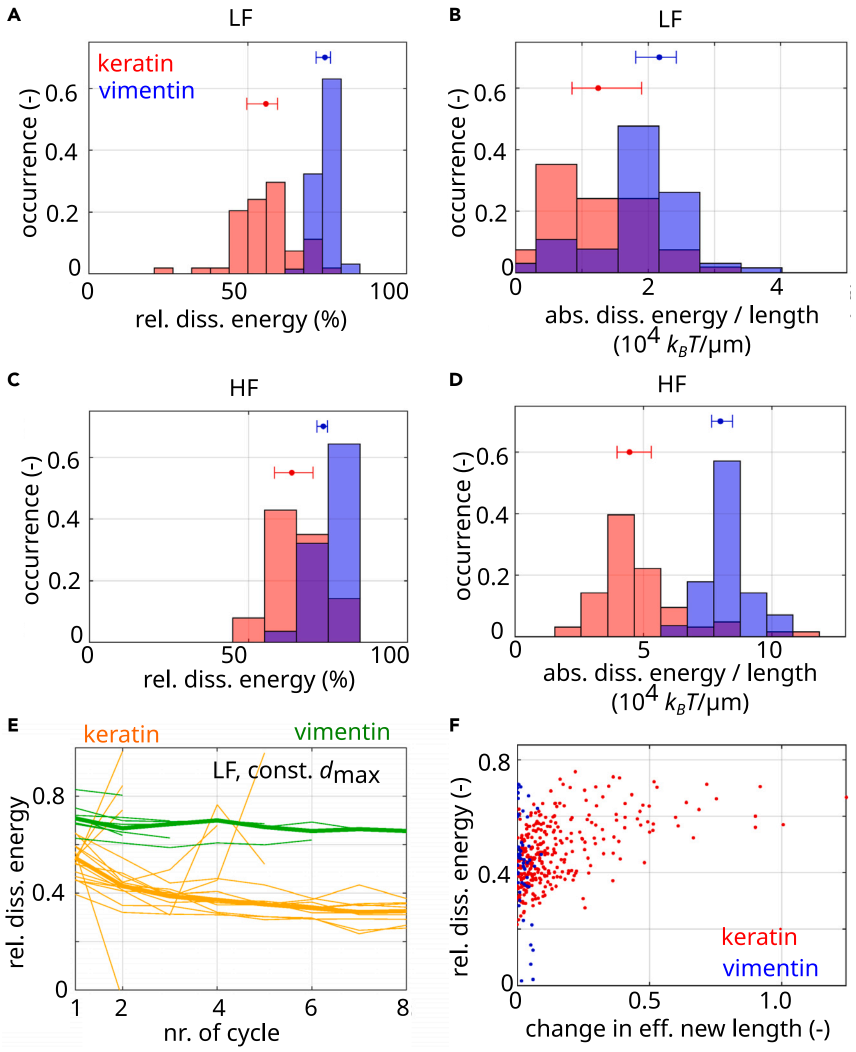

Figure 6. Keratin and vimentin filaments dissipate energy by different mechanisms

Dissipated energies of keratin (red) and vimentin filaments (blue) for the first stretching cycle.

(A and B) Relative dissipated energy (A) and absolute dissipated energy (B) per length of filaments stretched to the LF range.

(C and D) Relative dissipated energy (C) and absolute dissipated energy (D) per filament length when stretched to the HF range. Dots and whiskers indicate the median and the 25th and 75th percentiles of the distributions, respectively. Bin width of the histograms is determined with the Freedman-Diaconis rule.

(E) Relative dissipated energy plotted against the number of stretching cycles for keratin (orange) and vimentin (green) filaments stretched to a constant d_{max}.

(F) Relative dissipated energy of all cycles compared to $\Delta\varepsilon_e$.

sliding causes filament elongation and diminishes α helix unfolding. As the α helices stay intact, the stiffness of keratin filaments remains constant, independent of the loading history. Another consequence of the subunit sliding is keratin filament elongation. Contrarily, vimentin filaments retain their length but soften during repeated loading due to α helix unfolding.[18,19]

Remarkably, both intermediate filament protein types possess the same secondary structure. The mechanical differences are routed in variations in the primary protein

structure, i.e., the amino acid sequence. Differing charge and hydrophobicity patterns lead to different interactions within the filaments and cause the very distinct mechanical behaviors.[16] Specifically, a very distinct charge pattern of the vimentin monomer causes a tight arrangement, called compaction, of the vimentin tetramers within the filament.[23,24] We hypothesize that this compaction of vimentin filaments and stronger electrostatic and hydrophobic interactions within the filaments inhibit subunit sliding so that the α helix unfolding is energetically favorable with respect to subunit sliding. Keratin filaments do not exhibit this specific charge pattern necessary for compaction[3,34,35] so that subunits can slide[16,32,36] based on a so-called protofilament structure.[37–39] For keratin, we find that an analogy to the sliding mechanism of atoms within a metal: due to the delocalized electrons, layers of atom cores can slide within the periodic lattice of the crystal, allowing metals to change their length but retain their stiffness and to dissipate energy.[29] In the case of keratin intermediate filaments, subunits slide along one another other and can rebind due to the periodic structure of the filament, which results in a constant stiffness and elongation, as well as energy dissipation. Thus, one may claim that keratin filaments mimic a biological metal-like response to mechanical stress. In contrast to keratin, we speculate that the stretching mechanism occuring in vimentin filaments might be analogous to the stretching of a double-network hydrogel showing a Mullins effect[30]: the tightly connected protofilaments correspond to the covalently cross-linked polymers ensuring stability of the gel, and the repeated breakage of bonds within the α helix or random coil corresponds to the breakage of ionic cross-links within the hydrogel, which dissipate energy. These analogies show that the material properties of the biopolymers studied here correspond to either hard or soft, non-biological matter properties. Consequently, our findings point toward sustainable, biological materials with properties similar to non-biological materials.

Thus, the differential expression of intermediate filament proteins might be a way for cells to ensure that filaments either keep their original stiffness (keratin) or their original length (vimentin). We speculate that a constant stiffness during repeated loading can be a vital property for specific cell types such as skin cells so that they keep a constant pressure against the same applied force but are flexible in their length. A constant filament length might be desirable for more motile cells so that cells or part of the cell are protected from elongation. A prominent example is the vimentin "cage" found surrounding the nucleus of cells.[40,41] This is in line with the expression of vimentin in motile cells.[42] Additionally, we find that vimentin dissipates considerably more energy even after repeated loading. Thus, motile cells might rather express vimentin instead of keratin to dissipate more energy caused by deformation due to the cellular movement. Similarly, the properties of vimentin filaments and their networks might be important during intracellular transport of large organelles: vimentin filaments form a dense network with a small mesh size, which needs to be deformable without permanently changing its structure, when cargo is transported through.

To conclude, keratin filaments show exactly the behavior one would expect for a metal-like material, whereas the behavior of the vimentin filaments resembles an elastomer that shows a Mullins effect. The keratin filaments extend as we cycle them (Figures 2B and 2C) but keep the same stiffness (Figures 3A and 3B). The vimentin filaments are modified during the first few cycles and then maintain their softer stiffness (Figures 3A and 3B) while not changing their length (Figures 2B and 2C). Note also that the loops in keratin in Figure 2A are identical but shifted sideways, which indicates plasticity, while the loops for vimentin are completely

CellPress
OPEN ACCESS

different, showing that the material must be changing its molecular structure. Our findings foster the idea of differential expression of intermediate filament proteins as a tool for cells to adapt their mechanical properties to their surrounding environment: after repeated loading, keratin filaments elongate but exhibit a constant stiffness, while vimentin filaments retain their length and soften—although both filament types consist of monomers with the same secondary structure. We propose that weaker interaction strengths within keratin filaments than within vimentin filaments cause these distinct behaviors because they allow for subunit sliding and thereby protect the α helices within keratin intermediate filaments from unfolding. Interestingly, independent of the interaction strength within the two different filament types, both may act as cellular shock absorbers, as they dissipate a major part of the input energy. Yet, the mechanism by which energy is dissipated is completely different and relies on internal viscous friction for keratin filaments and non-equilibrium unfolding of α helices for vimentin filaments. One may speculate that the results could serve as a blueprint to design "smart," switchable, and degradable synthetic materials in the future.

EXPERIMENTAL PROCEDURES

The experimental procedures are described in the supplemental information.

Resource availability

Lead contact

Further information and requests for resources should be directed to and will be fulfilled by the lead contact, Sarah Köster (sarah.koester@uni-goettingen.de).

Materials availability

This study did not generate new unique reagents.

Data and code availability

All code is included in the supplemental information. The data have been deposited at GRO.data under https://doi.org/10.25625/LUYSTB and are publicly available as of the date of publication.

SUPPLEMENTAL INFORMATION

Supplemental information can be found online at https://doi.org/10.1016/j.matt.2023.04.014.

ACKNOWLEDGMENTS

We thank S. Bauch for the purification of the proteins. We are grateful for fruitful discussions with J. Kraxner, A.V. Schepers, and D.A. Weitz. The work was financially supported by the European Research Council (ERC; grant no. CoG 724932) and the Studienstiftung des deutschen Volkes e.V.

AUTHOR CONTRIBUTIONS

Conceptualization, S. Köster; methodology, C.L., J.F., S. Klumpp, and S. Köster; software: C.L. and S. Klumpp; formal analysis: C.L., J.F.; investigation, C.L. and J.F.; writing – original draft, C.L. and S. Köster; writing – review & editing, all authors; supervision, R.W.S., S. Klumpp, and S. Köster; funding acquisition, C.L. and S. Köster.

DECLARATION OF INTERESTS

The authors declare no competing interests.

Received: January 3, 2023
Revised: March 22, 2023
Accepted: April 24, 2023
Published: May 22, 2023

REFERENCES

1. Huber, F., Boire, A., López, M.P., and Koenderink, G.H. (2015). Cytoskeletal crosstalk: when three different personalities team up. Curr. Opin. Cell Biol. *32*, 39–47. https://doi.org/10.1016/j.ceb.2014.10.005.
2. Wu, H., Shen, Y., Sivagurunathan, S., Weber, M.S., Adam, S.A., Shin, J.H., Fredberg, J.J., Medalia, O., Goldman, R., and Weitz, D.A. (2022). Vimentin intermediate filaments and filamentous actin form unexpected interpenetrating networks that redefine the cell cortex. Proc. Natl. Acad. Sci. USA *119*. e2115217119. https://doi.org/10.1073/pnas.2115217119.
3. Herrmann, H., Häner, M., Brettel, M., Ku, N.O., and Aebi, U. (1999). Characterization of distinct early assembly units of different intermediate filament proteins. J. Mol. Biol. *286*, 1403–1420. https://doi.org/10.1006/jmbi.1999.2528.
4. Herrmann, H., Hesse, M., Reichenzeller, M., Aebi, U., and Magin, T.M. (2003). Functional complexity of intermediate filament cytoskeletons: from structure to assembly to gene ablation. Int. Rev. Cytol. *223*, 83–175. https://doi.org/10.1016/S0074-7696(05)23003-6.
5. Omary, M.B. (2009). “IF-pathies”: a broad spectrum of intermediate filament–associated diseases. J. Clin. Invest. *119*, 1756–1762. https://doi.org/10.1172/JCI39894.
6. Block, J., Schroeder, V., Pawelzyk, P., Willenbacher, N., and Köster, S. (2015). Physical properties of cytoplasmic intermediate filaments. Biochim. Biophys. Acta *1853*, 3053–3064. https://doi.org/10.1016/j.bbamcr.2015.05.009.
7. Franke, W.W., Grund, C., Kuhn, C., Jackson, B.W., and Illmensee, K. (1982). Formation of cytoskeletal elements during mouse embryogenesis: III. primary mesenchymal cells and the first appearance of vimentin filaments. Differentiation. *23*, 43–59. https://doi.org/10.1111/j.1432-0436.1982. tb01266.x.
8. Paranko, J., Kallajoki, M., Pelliniemi, L.J., Lehto, V.-P., and Virtanen, I. (1986). Transient coexpression of cytokeratin and vimentin in differentiating rat sertoli cells. Dev. Biol. *117*, 35–44. https://doi.org/10.1016/0012-1606(86)90345-3.
9. Paranko, J., and Virtanen, I. (1986). Epithelial and mesenchymal cell differentiation in the fetal rat genital ducts: changes in the expression of cytokeratin and vimentin type of intermediate filaments and desmosomal plaque proteins. Dev. Biol. *117*, 135–145. https://doi.org/10.1016/0012-1606(86)90356-8.
10. Pagan, R., Llobera, M., and Vilaró, S. (1995). Epithelial-mesenchymal transition in cultured neonatal hepatocytes. Hepatology *21*, 820–831. https://doi.org/10.1016/0270-9139(95)90537-5.
11. Pagan, R., Martín, I., Alonso, A., Llobera, M., and Vilaró, S. (1996). Vimentin filaments follow the preexisting cytokeratin network during epithelial–mesenchymal transition of cultured neonatal rat hepatocytes. Exp. Cell Res. *222*, 333–344. https://doi.org/10.1006/excr.1996.0043.
12. Thiery, J.P., and Sleeman, J.P. (2006). Complex networks orchestrate epithelial–mesenchymal transitions. Nat. Rev. Mol. Cell Biol. *7*, 131–142. https://doi.org/10.1038/nrm1835.
13. Fortier, A.-M., Asselin, E., and Cadrin, M. (2013). Keratin 8 and 18 loss in epithelial cancer cells increases collective cell migration and cisplatin sensitivity through caudin1 up-regulation. J. Biol. Chem. *288*, 11555–11571. https://doi.org/10.1074/jbc.M112.428920.
14. Lim, H.Y.G., Alvarez, Y.D., Gasnier, M., Wang, Y., Tetlak, P., Bissiere, S., Wang, H., Biro, M., and Plachta, N. (2020). Keratins are asymmetrically inherited fate determinants in the mammalian embryo. Nature *585*, 404–409. https://doi.org/10.1038/s41586-020-2647-4.
15. Cheng, F., and Eriksson, J.E. (2017). Intermediate filaments and the regulation of cell motility during regeneration and wound healing. Cold Spring Harbor Perspect. Biol. *9*, a022046. https://doi.org/10.1101/cshperspect.a022046.
16. Lorenz, C., Forsting, J., Schepers, A.V., Kraxner, J., Bauch, S., Witt, H., Klumpp, S., and Köster, S. (2019). Lateral subunit coupling determines intermediate filament mechanics. Phys. Rev. Lett. *123*, 188102. https://doi.org/10.1103/PhysRevLett.123.188102.
17. Block, J., Witt, H., Candelli, A., Peterman, E.J.G., Wuite, G.J.L., Janshoff, A., and Köster, S. (2017). Nonlinear loading-rate-dependent force response of individual vimentin intermediate filaments to applied strain. Phys. Rev. Lett. *118*, 048101. https://doi.org/10.1103/PhysRevLett.118.048101.
18. Block, J., Witt, H., Candelli, A., Danes, J.C., Peterman, E.J.G., Wuite, G.J.L., Janshoff, A., and Köster, S. (2018). Viscoelastic properties of vimentin originate from nonequilibrium conformational changes. Sci. Adv. *4*, eaat1161. https://doi.org/10.1126/sciadv.aat1161.
19. Forsting, J., Kraxner, J., Witt, H., Janshoff, A., and Köster, S. (2019). Vimentin intermediate filaments undergo irreversible conformational changes during cyclic loading. Nano Lett. *19*, 7349–7356. https://doi.org/10.1021/acs.nanolett.9b02972.
20. Herrmann, H., Häner, M., Brettel, M., Müller, S.A., Goldie, K.N., Fedtke, B., Lustig, A., Franke, W.W., and Aebi, U. (1996). Structure and assembly properties of the intermediate filament protein vimentin: the role of its head, rod and tail domains. J. Mol. Biol. *264*, 933–953. https://doi.org/10.1006/jmbi.1996.0688.
21. Kirmse, R., Portet, S., Mücke, N., Aebi, U., Herrmann, H., and Langowski, J. (2007). A quantitative kinetic model for the in vitro assembly of intermediate filaments from tetrameric vimentin. J. Biol. Chem. *282*, 18563–18572. https://doi.org/10.1074/jbc.M701063200.
22. Hosford, W.F. (2005). Mechanical Behavior of Materials (Cambridge University Press).
23. Premchandar, A., Kupniewska, A., Tarnowski, K., Mücke, N., Mauermann, M., Kaus-Drobek, M., Edelman, A., Herrmann, H., and Dadlez, M. (2015). Analysis of distinct molecular assembly complexes of keratin k8 and k18 by hydrogen–deuterium exchange. J. Struct. Biol. *192*, 426–440. https://doi.org/10.1016/j.jsb.2015.10.001.
24. Premchandar, A., Mücke, N., Poznański, J., Wedig, T., Kaus-Drobek, M., Herrmann, H., and Dadlez, M. (2016). Structural dynamics of the vimentin coiled-coil contact regions involved in filament assembly as revealed by hydrogen-deuterium exchange. J. Biol. Chem. *291*, 24931–24950. https://doi.org/10.1074/jbc.M116.748145.
25. Eldirany, S.A., Ho, M., Hinbest, A.J., Lomakin, I.B., and Bunick, C.G. (2019). Human keratin 1/10-1b tetramer structures reveal a knob-pocket mechanism in intermediate filament assembly. EMBO J. *38*, e100741. https://doi.org/10.15252/embj.2018100741.
26. Bartczak, Z., and Galeski, A. (2010). Plasticity of semicrystalline polymers. Macromol. Symp. *294*, 67–90. https://doi.org/10.1002/masy.201050807.
27. Qin, Z., Kreplak, L., and Buehler, M.J. (2009). Hierarchical structure controls nanomechanical properties of vimentin intermediate filaments. PLoS One *4*, e7294. https://doi.org/10.1371/journal.pone.0007294.
28. Chou, C.-C., and Buehler, M.J. (2012). Structure and mechanical properties of human trichocyte keratin intermediate filament protein. Biomacromolecules *13*, 3522–3532. https://doi.org/10.1021/bm301254u.
29. Hofmann, P. (2015). Solid State Physics: An Introduction (John Wiley & Sons).

CellPress
OPEN ACCESS

30. Sun, J.-Y., Zhao, X., Illeperuma, W.R.K., Chaudhuri, O., Oh, K.H., Mooney, D.J., Vlassak, J.J., and Suo, Z. (2012). Highly stretchable and tough hydrogels. Nature *489*, 133–136. https://doi.org/10.1038/nature11409.

31. Zhang, W., Liu, X., Wang, J., Tang, J., Hu, J., Lu, T., and Suo, Z. (2018). Fatigue of double network hydrogels. Eng. Fract. Mech. *187*, 74–93. https://doi.org/10.1016/j.engfracmech.2017.10.018.

32. Kreplak, L., Bär, H., Leterrier, J.F., Herrmann, H., and Aebi, U. (2005). Exploring the mechanical behavior of single intermediate filaments. J. Mol. Biol. *354*, 569–577. https://doi.org/10.1016/j.jmb.2005.09.092.

33. Herrmann, H., Bär, H., Kreplak, L., Strelkov, S.V., and Aebi, U. (2007). Intermediate filaments: from cell architecture to nanomechanics. Nat. Rev. Mol. Cell Biol. *8*, 562–573. https://doi.org/10.1038/nrm2197.

34. Winheim, S., Hieb, A.R., Silbermann, M., Surmann, E.-M., Wedig, T., Herrmann, H., Langowski, J., and Mücke, N. (2011). Deconstructing the late phase of vimentin assembly by total internal reflection fluorescence microscopy (TIRFM). PLoS One *6*, e19202. https://doi.org/10.1371/journal.pone.0019202.

35. Lichtenstern, T., Mücke, N., Aebi, U., Mauermann, M., and Herrmann, H. (2012). Complex formation and kinetics of filament assembly exhibited by the simple epithelial keratins K8 and K18. J. Struct. Biol. *177*, 54–62. https://doi.org/10.1016/j.jsb.2011.11.00.

36. Goldman, R.D., Grin, B., Mendez, M.G., and Kuczmarski, E.R. (2008). Intermediate filaments: versatile building blocks of cell structure. Curr. Opin. Cell Biol. *20*, 28–34. https://doi.org/10.1016/j.ceb.2007.11.003.

37. Aebi, U., Fowler, W.E., Rew, P., and Sun, T.T. (1983). The fibrillar substructure of keratin filaments unraveled. J. Cell Biol. *97*, 1131–1143. https://doi.org/10.1083/jcb.97.4.1131.

38. Parry, D.A., Marekov, L.N., and Steinert, P.M. (2001). Subfilamentous protofibril structures in fibrous proteins. J. Biol. Chem. *276*, 39253–39258. https://doi.org/10.1074/jbc.m104604200.

39. Goldie, K.N., Wedig, T., Mitra, A.K., Aebi, U., Herrmann, H., and Hoenger, A. (2007). Dissecting the 3-d structure of vimentin intermediate filaments by cryo-electron tomography. J. Struct. Biol. *158*, 378–385. https://doi.org/10.1016/j.jsb.2006.12.007.

40. Patteson, A.E., Vahabikashi, A., Pogoda, K., Adam, S.A., Mandal, K., Kittisopikul, M., Sivagurunathan, S., Goldman, A., Goldman, R.D., and Janmey, P.A. (2019). Vimentin protects cells against nuclear rupture and DNA damage during migration. J. Cell Biol. *218*, 4079–4092. https://doi.org/10.1083/jcb.201902046.

41. Pogoda, K., Byfield, F., Deptuła, P., Cieśluk, M., Suprewicz, Ł., Skłodowski, K., Shivers, J.L., van Oosten, A., Cruz, K., Tarasovetc, E., et al. (2022). Unique role of vimentin networks in compression stiffening of cells and protection of nuclei from compressive stress. Nano Lett. *22*, 4725–4732. https://doi.org/10.1021/acs.nanolett.2c00736.

42. Lavenus, S.B., Tudor, S.M., Ullo, M.F., Vosatka, K.W., and Logue, J.S. (2020). A flexible network of vimentin intermediate filaments promotes migration of amoeboid cancer cells through confined environments. J. Biol. Chem. *295*, 6700–6709. https://doi.org/10.1074/jbc.RA119.011537.

Matter, Volume *6*

Supplemental information

Keratin filament mechanics and energy dissipation are determined by metal-like plasticity

Charlotta Lorenz, Johanna Forsting, Robert W. Style, Stefan Klumpp, and Sarah Köster

Supplemental figures

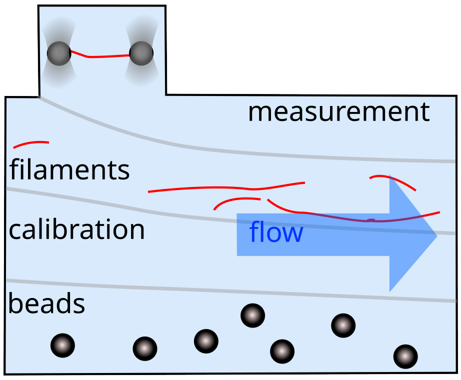

Figure S1: **Sketch of the microfluidic setup combined with optical tweezers to stretch single intermediate filaments.** The thin gray lines denote the borders of the different solutions flowing side-by-side in a laminar manner, without turbulent mixing.

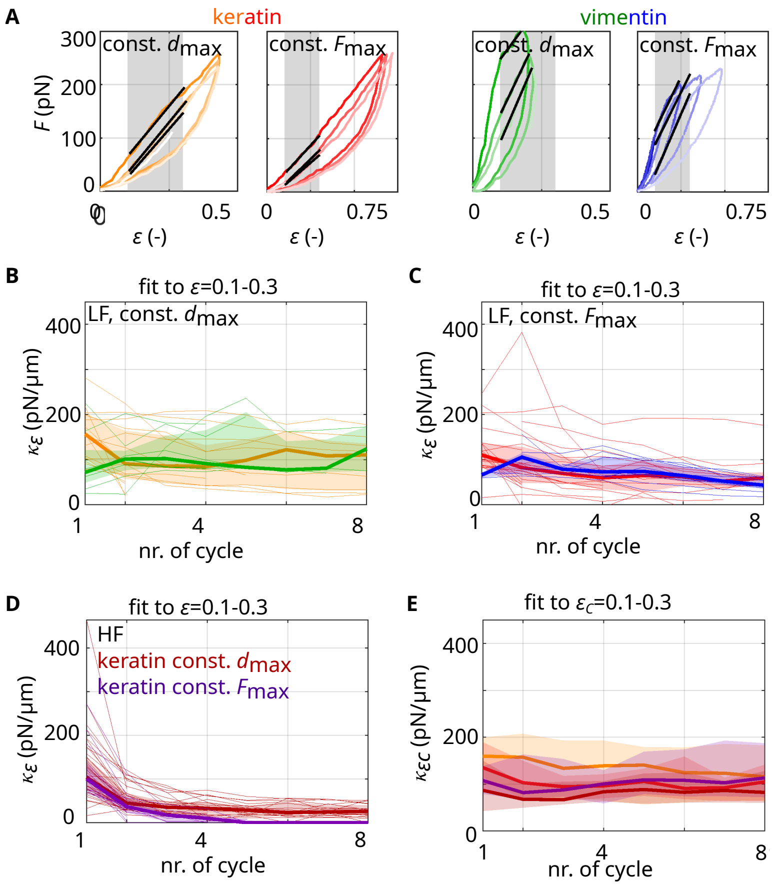

Figure S2: **Keratin filaments retain their stiffness upon repeated loading**, related to Fig. 3. (A) Typical experimental data sets of keratin (orange, red) and vimentin (green, blue) filaments stretched three times to a constant $d_{\max}$ (orange, green) and to a constant $F_{\max}$ (red, blue) including fits to $\varepsilon = 0.1 - 0.3$ (gray shaded area, solid black lines); same data as shown in Fig. 2A. (B,C) Keratin (orange, red) and vimentin (green, blue) filament stretching stiffness κ_ε resulting from the fits shown in a. (B) constant $d_{\max}$ with $F_{\max}$ in the LF range; (C) constant $F_{\max}$ in the LF range. (D) Keratin filament stiffness κ_ϵ derived from linear fits to $\varepsilon = 0.1 - 0.3$ for keratin filaments stretched to the HF range with a constant $d_{\max}$ (dark red) or to a constant $F_{\max}$ (purple). (E) Corrected $\kappa_{\epsilon c}$ for keratin filaments for all conditions studied: constant $d_{\max}$ with $F_{\max}$ in the LF range (orange), constant $F_{\max}$ in the LF range (red), constant $d_{\max}$ with a $F_{\max}$ in the HF range (dark red) and constant $F_{\max}$ in the HF range (purple). (B-E) Thick lines show the median and shading indicates the area between the 25th and 75th percentile of the distributions per cycle.

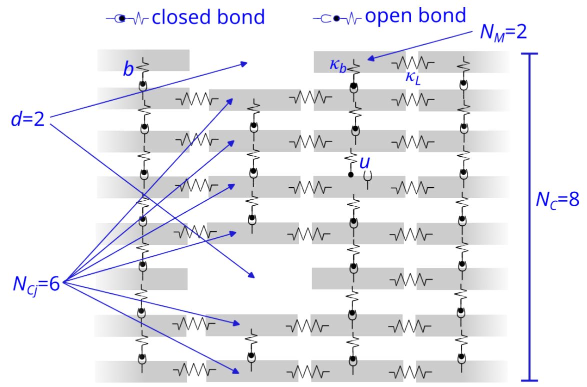

Figure S3: **Sketch of the model describing subunit sliding**, related to Fig. 4. The parameters are defined in the Supplemental experimental methods.

Supplemental tables

Variable	Meaning	Value
ΔG	Energy difference between a laterally bound and unbound subunit	6
κ_b	Stiffness of a lateral bond between two subunits	4.6
κ_L	Stiffness of all linkers between subunits	60
N_C	Number of subunits within a filament	8
N_F	Number of unit-length filaments within a filament	100
N_M	Number of monomers within a subunit	2
N_P	Number of parallel monomers in a unit-length filament	16
p_S	Probability that a slid dimer binds to the next dimer	10%
v	Loading speed	10000

Table S1: **Dimensionless parameters and values for simulation of single stretched keratin filaments.**

Variable	Meaning	Value
ΔG	Energy difference between a laterally bound and unbound subunit	2
κ_α	Stiffness of a lateral bond between two subunits	5.5
κ_β	Stiffness of all linkers between subunits	5.5
κ_L	Stiffness of all linkers between subunits	100
N_F	Number of unit-length filaments within a filament	100
N_P	Number of parallel monomers in a unit-length filament	32
v	Loading speed	2000

Table S2: **Dimensionless parameters and values for simulation of single stretched vimentin filaments.**

Supplemental experimental procedures

Materials and methods

Protein is expressed, labeled and dialyzed as described in Ref. 1. After assembly, keratin filaments are diluted 1:10 with keratin assembly buffer (10 mM TRIS, pH 7.5)[2–4] and stored at 37°C for up to two days. Vimentin filaments are stored in vimentin assembly buffer (100 mM KCl, 2 mM phosphate buffer, pH 7.5)[5,6] at 4°. For measurements with the optical tweezers setup (LUMICKS, Amsterdam, Netherlands), we load a four-channel microfluidic chip. Two maleimide-coated polystyrene beads (Kisker Biotech, Steinfurt, Germany) are captured with the optical tweezers from the beads channel and calibrated via the thermal noise spectrum. All filaments are stretched in vimentin assembly buffer, which corresponds to the high-ionic strength buffer condition used in Ref. 1.

All data analysis and image processing is carried out with self-written MatLab codes.

Simulations

Single keratin filaments under tension are modeled based on Refs. 1,7,8. One unit length filament (ULF) has N_P monomers, which are arranged in parallel and all have the same length before loading. The monomers can be divided into N_C N_M-mers, i.e., a keratin filament[9] has $N_C = 8$ dimers with $N_M = 2$. We refer to these N_M-mers as "subunits", which have the ability to slide with respect to each other. When subunits slide, the bonds between them open and re-form at the neighboring periodic position in the direction of the applied force as sketched in Fig. 4B. In accordance with previous findings[10,11] we assume that interactions within subunits are strong compared to interactions between these subunits so that the interactions within subunits, i.e. between monomers, do not break. Yet, the model is still valid if subunits are assumed to be

equivalent monomers. Once a dimer has slid, it either pulls all dimers in a protofilament with it or it leaves a defect, i.e., an empty dimer as sketched in Fig. 4A, B.

We model the interaction between subunits by a spring with a spring constant κ_b and by an element that can open into an unbound state u when force is applied and can close again into a bound state b, when the connection to a new dimer forms. A sketch of the model is shown in Fig. S3. B_j is the number of intact lateral bonds between dimers in the jth ULF. The longitudinal interaction between subunits forming a protofilament is described with a spring constant κ_L.

Thus, the spring constant of the jth ULF is:

$$\kappa_j = N_C \left(\frac{1}{\kappa_L} + \frac{1}{B_j \kappa_b} \right)^{-1} .$$

The spring constant of the entire filament is calculated by $\kappa_F = 1/\left(\sum_{j=1}^{N_F} 1/\kappa_j\right)$, where N_F is the number of ULFs in a filament. Similar to Ref. 7, we neglect viscous and entropic contributions for simplicity.

We assume that the sliding of the subunits is a close-to-equilibrium process under force. The equilibrium reaction constant K_{eq} for subunits to unbind is:

$$K_{eq} = \frac{r^{b\to u}}{r^{u\to b}} = \exp(-\Delta G/(k_B T)) = 1/\gamma\,, \tag{1}$$

where we define $\gamma = \exp(\Delta G/(k_B T))$.

The force is distributed among the number of independent subunits N_C. The effect of the force on the rates is distributed between the binding and unbinding rates with a load distribution factor[12,13] θ, which ensures detailed balance and thus that the model leads to a thermodynamic equilibrium state in the non-driven limit.

The force $\phi = F/F_b$ is dimensionless and scaled to the force F_b which is required to open a bond between two subunits. The time τ is dimensionless and related to the time t with the zero-force reaction rate from a subunit binding to another subunit to the unbound state $r_0^{b\to u}$ by $\tau = r_0^{b\to u} t$. We assume Bell-Evans kinetics[14], so that the bound to unbound transition rate is:

$$r_{B_j}^{b\to u} = B_j r_0^{b\to u} \exp\left(\frac{\theta\phi}{B_j}\right) . \tag{2}$$

In this study, we set $\theta = 1$, so only the unbinding rate is affected by the force. We assume that there is no restoring force acting on subunits which rebind to a neighboring subunit in the direction of loading as sketched in Fig. 4A, B. Yet, subunits can unbind without rebinding to the next subunit in the direction of loading because the disposition of the dimer is not sufficient to displace the subunit up to the periodic position. To describe the rebinding event to a new subunit or rebinding to the original subunit, we calculate the rebinding rate $r_{B_j}^{u\to b}$ depending on the force distributed on the N_{Cj} subunits of the jth ULF (if the ULF possesses d defects, the number of subunits $N_{Cj} = N_C - d$):

$$r_{B_j}^{u\to b} = r_0^{b\to u} \gamma \exp\left(\frac{-(1-\theta)\phi}{N_{Cj}}\right) \tag{3}$$

We assume that the filament possesses 50 defects before it is stretched to approximate the experimental data. From these rates, we calculate the probability P_{B_j} that a certain number of subunits is bound laterally to another subunit. The dynamics of this quantity are given by a master equation which we simulate using the Gillespie algorithm:

$$\begin{aligned} \frac{dP_{B_j}}{dt} &= r_{B_j+1}^{b\to u} P_{B_j+1} + r_{B_j-1}^{u\to b} P_{B_j-1} \\ &\quad -(r_{B_j}^{b\to u} + r_{B_j}^{u\to b}) P_{B_j} . \end{aligned} \tag{4}$$

When a subunit unbinds, the ULF extends by ΔL and with a probability of $p_S = 10\%$ the dimer slides and can bind to a new dimer to approximate the experimental data. If the filament is not stretched further than an effective elongation of 0.15, dimers do not slide and only unbind

and rebind at the same position. We calculate the effective elongation by analyzing the extension of the filament during a retraction cycle at a force of 0.5 (arbitrary units).

To make the simulation run dimensionless, we normalize $\lambda = \Delta L/L_1$, where L_1 is a characteristic length of a unit-length filament. Thus, in this case, the extension of the jth ULF λ_j is:

$$\lambda_j = \begin{cases} 0 & \text{if } B_j > 0 \\ 1 & \text{if } B_j = 0. \end{cases} \tag{5}$$

The total extension of the filament then is $\lambda_{tot} = \sum_{i=1}^{N_F} \lambda_j$. Since the optical traps pull on the filament with a constant velocity v, the end-to-end distance is $x(t) = vt$. The force on the filament becomes

$$\phi = \kappa_F(x - \lambda_{tot})\,. \tag{6}$$

To simulate the data shown in Fig. 5, we use the parameters provided in Table S1.

The simulation of single stretched vimentin filaments is taken from Ref. 7 with the parameters provided in Table S2.

To convert these parameters into real values with actual units, fitting of the resulting force-strain curves would be necessary as shown in the Supplemental Material of Ref. 1.

Supplemental references

[1] Lorenz, C., Forsting, J., Schepers, A. V., Kraxner, J., Bauch, S., Witt, H., Klumpp, S., and Köster, S. (2019). Lateral subunit coupling determines intermediate filament mechanics. Phys. Rev. Lett. 123, 188102. 10.1103/PhysRevLett.123.188102.

[2] Coulombe, P. A. and Fuchs, E. (1990). Elucidating the early stages of keratin filament assembly. J. Cell Biol. 111, 153–169. 10.1083/jcb.111.1.153.

[3] Herrmann, H., Häner, M., Brettel, M., O Ku, N., and Aebi, U. (1999). Characterization of distinct early assembly units of different intermediate filament proteins. J. Mol. Biol. 286, 1403–20. 10.1006/jmbi.1999.2528.

[4] Herrmann, H., Wedig, T., Porter, R. M., Lane, E. B., and Aebi, U. (2002). Characterization of early assembly intermediates of recombinant human keratins. J. Struct. Biol. 137, 82–96. 10.1006/jsbi.2002.4466.

[5] Mücke, N., Wedig, T., Bürer, A., Marekov, L. N., Steinert, P. M., Langowski, J., Aebi, U., and Herrmann, H. (2004). Molecular and biophysical characterization of assembly-starter units of human vimentin. J. Mol. Biol. 340, 97–114. 10.1016/j.jmb.2004.04.039.

[6] Kretsinger, R. H., Uversky, V., and Permyakov, E. (2013). Encyclopedia of Metalloproteins (Springer New York).

[7] Block, J., Witt, H., Candelli, A., Danes, J. C., Peterman, E. J., Wuite, G. J., Janshoff, A., and Köster, S. (2018). Viscoelastic properties of vimentin originate from nonequilibrium conformational changes. Sci. Adv. 4, eaat1161. 10.1126/sciadv.aat1161.

[8] Forsting, J., Kraxner, J., Witt, H., Janshoff, A., and Köster, S. (2019). Vimentin intermediate filaments undergo irreversible conformational changes during cyclic loading. Nano Lett. 19, 7349–7356. 10.1021/acs.nanolett.9b02972.

[9] Herrmann, H., Häner, M., Brettel, M., Müller, S. A., Goldie, K. N., Fedtke, B., Lustig, A., Franke, W. W., and Aebi, U. (1996). Structure and assembly properties of the intermediate filament protein vimentin: the role of its head, rod and tail domains. J. Mol. Biol. 264, 933–953. 10.1006/jmbi.1996.0688.

[10] Premchandar, A., Kupniewska, A., Tarnowski, K., Mücke, N., Mauermann, M., Kaus-Drobek, M., Edelman, A., Herrmann, H., and Dadlez, M. (2015). Analysis of distinct molecular assembly complexes of keratin k8 and k18 by hydrogen–deuterium exchange. J. Struct. Biol. 192, 426 – 440. 10.1016/j.jsb.2015.10.001.

[11] Premchandar, A., Mücke, N., Poznański, J., Wedig, T., Kaus-Drobek, M., Herrmann, H., and Dadlez, M. (2016). Structural dynamics of the vimentin coiled-coil contact regions involved in filament assembly as revealed by hydrogen-deuterium exchange. J. Biol. Chem. 291, 24931–24950. 10.1074/jbc.M116.748145.

[12] Kolomeisky, A. (2015). Motor Proteins and Molecular Motors (CRC Press).
[13] Fisher, M. E. and Kolomeisky, A. B. (1999). The force exerted by a molecular motor. Proc. Natl. Acad. Sci. USA 96, 6597–6602. 10.1073/pnas.96.12.6597.
[14] Bell, G. (1978). Models for the specific adhesion of cells to cells. Science 200, 618–62. 10.1126/science.347575.
